# Understanding the relationship between prevalence of microfilariae and antigenaemia using a model of lymphatic filariasis infection

**DOI:** 10.1093/trstmh/trv096

**Published:** 2016-01-28

**Authors:** Michael A. Irvine, Sammy M. Njenga, Shamini Gunawardena, Claire Njeri Wamae, Jorge Cano, Simon J. Brooker, T. Deirdre Hollingsworth

**Affiliations:** a School of Life Sciences, University of Warwick, Coventry, CV4 7AL, UK; b Kenya Medical Research Institute (KEMRI), P.O. Box 54840, Post Code 00200, Nairobi, Kenya; c School of Health Sciences, Mount Kenya University, P.O. Box 342-01000, Thika, Kenya; d London School of Hygiene & Tropical Medicine, Keppel Street, London WC1E 7HT, UK; e School of Health Sciences, Mount Kenya University, P.O. Box 342-01000, Thika, Kenya; f Mathematics Institute, University of Warwick, Coventry, CV4 7AL, UK

**Keywords:** Antigenaemia, Elimination programme, Lymphatic filariasis, Microfilaraemia, Prevalence surveys

## Abstract

**Background:**

Lymphatic filariasis is a debilitating neglected tropical disease that affects impoverished communities. Rapid diagnostic tests of antigenaemia are a practical alternative to parasitological tests of microfilaraemia for mapping and surveillance. However the relationship between these two methods of measuring burden has previously been difficult to interpret.

**Methods:**

A statistical model of the distribution of worm burden and microfilariae (mf) and resulting antigenaemic and mf prevalence was developed and fitted to surveys of two contrasting sentinel sites undergoing interventions. The fitted model was then used to explore the relationship in various pre- and post-intervention scenarios.

**Results:**

The model had good quantitative agreement with the data and provided estimates of the reduction in mf output due to treatment. When extrapolating the results to a range of prevalences there was good qualitative agreement with published data.

**Conclusions:**

The observed relationship between antigenamic and mf prevalence is a natural consequence of the relationship between prevalence and intensity of adult worms and mf production. The method described here allows the estimation of key epidemiological parameters and consequently gives insight into the efficacy of an intervention programme.

## Introduction

Lymphatic filariasis (LF) is a debilitating disease caused by parasitic infection of the lymph nodes.^1^ Although most cases displayed are asymptomatic, prolonged and high burden can lead to abnormal enlargement of body parts causing pain, severe disability and social stigma. There are currently global efforts to eliminate the disease as a public health problem by 2025 through the use of mass drug administration (MDA) with a combination of albendazole and either ivermectin (in onchocerciasis endemic areas) or diethylcarbamazine (in areas without onchocerciasis). The campaign has had a great number of successes, but problems and questions remain as to whether the set targets are achievable.^2^

One challenge facing the monitoring and evaluation of programmes is the use of different diagnostic tests. Transmission of the filarial worm is through mosquitoes; where microfilariae (mf) in an infected host's blood are ingested by the mosquito, and develop into infective larvae that are able to enter another susceptible host when the mosquito takes another blood meal. Therefore mf counts are a good tool for estimating not only the prevalence of infection, but also the mean mf load and infectious pool in a community.^3^ Microfilariae are counted in blood samples through either a blood smear or a counting chamber and therefore require expert parasitologists to detect. In addition, in Africa, where several million people are at-risk, measures can only be taken through blood samples at night, because of *Wucheria bancrofti*'s nocturnal periodicity. Sensitivity and specificity of the test are 97% and 100% respectively, although this is dependent upon mf load, blood volume and parasitological method.^4,5^

A simpler method of measuring prevalence of LF is an antigenic immunochromatographic test (ICT). The test relies upon detecting antigens secreted by adult worms in a blood sample. Due to its cost, fewer requirements for training and lack of necessity to take a blood sample at night, the ICT has become a popular alternative to mf testing.^6^ However, ICT prevalence cannot measure the infective pool due to the nonlinear correspondence between worm burden and mf output^7^ and, more importantly, the action of the MDA.^8^ The treatments being used in MDA primarily kill mf, with some additional effect on mortality and sterilisation of adult worms.^9^ Therefore, after a number of rounds of MDA there is a possibility that ICT prevalence could remain high whilst mf prevalence is very low, potentially leading to unnecessary rounds of MDA.

Given the different profiles of these two diagnostic methods, and a general shift from mf surveys to ICT surveys, it is important to understand the relationship between the two. However, there is currently a lack of good correspondence between the antigen and microfilaraemia prevalence.^10^ A recent study compared the ICT and mf prevalence in both pre- and post-intervention settings and found that no covariates could explain the discrepancy between the two and suggested that predicting mf prevalence from ICT prevalence remains an open problem.^10^

Here we propose that the observed prevalence of antigenaemia and mf is a consequence of the underlying distribution of adult worms and their mf output. This nonlinear relationship could explain the observed patterns, and be used to interpret surveillance data.

In order to test this hypothesis, we use standard assumptions about the distribution of adult worms and egg output to build a model of the resulting prevalence of antigenaemia and mf positivity. We fit the model to data from two contrasting sentinel sites. The first from Malindi, Kenya was conducted at the start of an intervention programme and has surveys conducted at baseline and for two rounds of MDA in 2002 to 2004. This is contrasted with a survey from Western Sri Lanka in the later stages of the MDA programme in 2004 and 2005. We then extrapolate the model to understand how the relationship would change for different baseline prevalences and compare the results to data from a recent review of the literature.^10^

## Materials and methods

### Model

**Figure 1.**
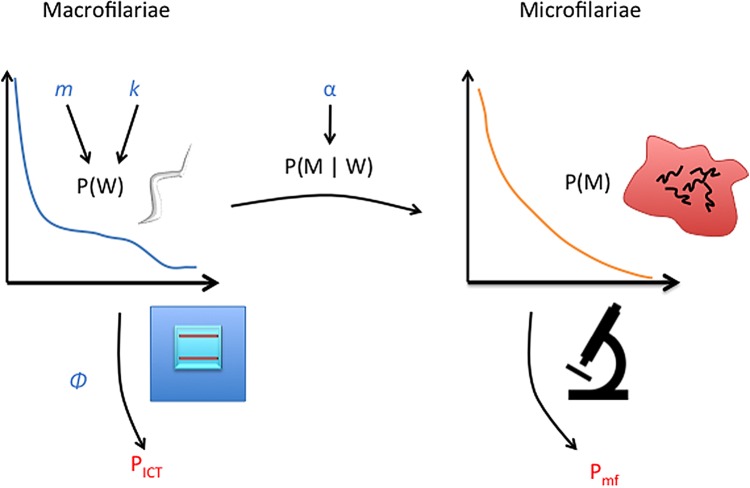
Overview of statistical model. The parameters mean worm burden *m* and aggregation of adult worms *k_w_* in the population are used to construct the probability of an individual having a given worm burden P(W). The microfilariae (mf) production and detection parameter *α* is used to informs the probability of a given mf count conditional on a given worm load P(M | W). These two probabilities are combined to produce the mf distribution in the population P(M). The prevalence of antigen positives *p_ICT_* are then based on the probability of a non-zero worm load with the test sensitivity *φ*. The prevalence of mf positives in the population are similarly calculated as the probability of an individual having a non-zero number of mf detected.

The model is based on an underlying distribution of adult worm loads in the population, with each individual having a probability of testing positive by each of the two tests based on their antigenaemia and mf output, leading to the resulting observed population prevalence by each test. The main idea is to construct the distribution of worm load in the population and the probability distribution of mf for an individual with a given worm load and use these to calculate the prevalence of antigenaemia and microfilaraemia in the population. A schematic of this is given in Figure 1.

The underlying distribution of adult worms in a population is known to be over dispersed. The distribution of worm burden, P(W), in the population is therefore modelled as negative binomial with mean worm burden *m* and aggregation parameter *k_w_*.^11^ The aggregation parameter controls the extent of the over dispersion of the worm population, with a highly aggregated distribution when *k_w_* is <1 and a more evenly distributed worm population for large *k_w_*. The ICT test detects if an individual is antigenaemic, based on antigens secreted by the adult worms.^12,13^ The test therefore is assumed to detect the presence of adult worms with a pre-defined sensitivity, *ϕ*. The probability of a random individual in the population receiving a positive result from the ICT test is therefore the probability of having a positive worm burden multiplied by the probability of detection according to the sensitivity of the test,

$$\;p_{ICT}=\phi P(W>0)=\phi \left(1-{\left(1+\frac{m}{k_{w}}\right)}^{-k_{w}}\right).$$

The mf prevalence is slightly more involved to derive as it not only depends upon the distribution of worms in the population, but also on the distribution of mf being produced by the adult worms. The production and detection of mf per adult worm is assumed to be constant throughout the population, a simplification of the density dependent assumptions in some models. We cannot independently estimate both the mf production rate and the sensitify of mf measurements, and we therefore use a single parameter for the production of detectable mf. Assuming a Poisson-count distribution of mf per adult worm, the probability of an individual with worm burden *w* having an mf count of *j* is therefore

$$P(M=j\;|\;W=w)=\frac{{\left(\alpha w\right)}^{j}}{\;j!}e^{-\alpha w}.$$

From this, the probability of a detectable mf burden, given an adult worm burden, w, is

P(M>0|W=w)=1−P(M=0|W=w).

To derive the prevalence of mf, we now need to calculate the probability that an individual is mf positive in this population. This is a combination of their probability of having a certain worm burden, w, and the probability that this worm burden results in detection.

$$\begin{array}{cc} \;p_{mf} & =P(M>0)=1-P(M=0)\\ & =1-\overset{\infty }{\underset{w=0}{\sum }}P(M=0|W=w)P(W=w). \end{array}$$

Substituting in the derived expressions for the probability of mf count given a worm burden and the probability of observing a particular worm burden produces a probability of mf count dependent upon the population parameters: mean worm burden, aggregation of worm burden in the population and rate of production and detection of mf. A positive mf test occurs if there is a non-zero measure of mf in a sample. The resulting probability of microfilaraemia is therefore

$$\;p_{mf}=1-\overset{\infty }{\underset{w=0}{\sum }}\frac{e^{-\alpha w}\Gamma (k_{w}+w)}{w!\Gamma (k_{w})}{\left(1+\frac{m}{k_{w}}\right)}^{-k-w}{\left(\frac{m}{k_{w}}\right)}^{w}.$$

The sensitivity of the ICT test, φ, is set according to literature estimates (97%), whilst the other model parameters are assumed to vary and hence were fitted to data. The model was validated by comparing the predicted probability of an individual being both ICT positive and mf negative with the proportion of individuals in the data. Using the literature-based estimate for the specificity of the ICT (*ψ)*, this probability can be calculated as 1-*p_mf_* – *ψ*(1-*p_ICT_*), with the specificity value taken as 98.4%.^14^

### Model fitting

In order to estimate the parameters of the derived relationship the model was fitted to two studies. The first is a study of two rounds of MDA using diethylcarbamazine combined with albendazole across four sentinel communities in Malindi, Kenya in the years 2002–2004 inclusive.^15^ Baseline blood samples were taken in all four communities before the first round of MDA in February 2002. Thereafter, blood samples were taken in March 2003 (post-MDA1) and July 2004 (post-MDA2). Both ICT and mf tests were performed on the same blood samples to provide an estimate for the prevalence of antigenaemia and microfilaraemia in the population. In order to retain statistical power, the results from the four communities were aggregated to provide a total number sampled in each survey of n=808.

Further analysis was conducted on a 2-year study in two districts in Western Sri Lanka during an MDA programme.^16^ Antigenaemia was measured through ICT and microfilaraemia measured through membrane filtration in the districts of Colombo and Gampaha. Measurements were taken after an MDA round in September 2004 and just prior to another MDA round in February 2005.

The model was fitted using a Bayesian Markov chain Monte Carlo (MCMC) framework. A likelihood was constructed assuming binomial sample error for the number of individuals testing positive in the total population of individuals surveyed. For a given population *n, with* the number of ICT positive individuals n_ICT,_ ICT negative individuals (n-n_ICT_), mf positive individuals n_mf_ and mf negative individuals (1-n_mf_), the log-likelihood is defined as

$$l=n_{ICT}\log\;p_{ICT}+(n-n_{ICT})\log(1-\;p_{ICT})+n_{mf}\log\;p_{mf}+(n-n_{mf})\log(1-\;p_{mf})$$

Exponential priors were assumed for all parameters of the model with means set to values from the literature *m*=1,α=0.2,*k*=0.1.^17,18^ The posterior was sampled using a Metropolis-Hastings sampler using 40 000 iterations, including 10 000 iterations for burn-in. The model was then fitted to each year when the survey was conducted. Fitting was performed using the PyMC statistical package in Python.^19^

### Immunochromatographic test–microfilariae relationship

The fit of the model to each setting along with the estimated posterior of parameter values was used to explore the credible relationship between antigenaemia and microfilaraemia. In each setting the relationship between the prevalence of mf and the prevalence of antigenaemia in a population in a given intervention regime was explored by first constructing a credible interval for the adult worm population parameters (*m,k­_w_*) and the mf production and detection rate (*α*) using the estimated posterior. The estimated 95% confidence range for each fit was used to produce an interval range for each parameter. These were then uniformly sampled and the calculated prevalence of mf and antigenaemia were plotted to produce a credible region for the relationship.

To understand the broad differences between the relationship in the pre- and post-intervention setting, a range of parameters were constructed based on known efficacies of drug regimens. The pre-intervention range of parameters was taken from the pre-intervention Kenyan 95% confidence interval range, with a smaller lower bound for the mean worm burden to account for areas with lower transmission than those observed in the baseline data. Current most effective drug regimens have a macrofilaricidal effect of 45% and a microfilaricidal effect of 95%.^12^ The mean worm burden upper and lower range was therefore reduced by 45% each and the mf output was reduced by 45% to construct the post-intervention range. These ranges for the parameters were then used to simulate the p_mf_ and p_ICT_ relationship.

## Results

The model was first fitted to the Kenya data set, to each survey year separately and the maximum a posteriori was calculated for all model parameters (Table 1). The baseline mf production is in close correspondence with literature estimates.^20^ The mf production and detection rate, mean worm burden and worm aggregation all reduce each year (0.25–0.16, 1.94–0.76 and 0.20–0.11). Both mf production and mean worm burden reducing suggest microfilariacidal and adulticidal effects of the MDA. Aggregation of worms also increases, which would be expected if drug coverage and efficacy were not evenly distributed in the population.

**Table 1.** Estimated parameters (with 95% CIs) for Poisson-dispersed mf model for 3 years the surveys were conducted in Malindi, Kenya

Year	mf detected in 100 μL blood per worm *α*	Mean worm burden *μ*	Worm aggregation *k_w_*
2002 (baseline)	0.254 (0.117–0.562)	1.943 (0.72–5.457)	0.205 (0.124–0.499)
2003	0.186 (0.063–0.414)	1.177 (0.508–3.796)	0.148 (0.081–0.337)
2004	0.161 (0.048–0.384)	0.756 (0.324–2.849)	0.11 (0.055–0.279)

mf: microfilariae.

The model was further fitted to a contrasting dataset from Sri Lanka where a number of years of intervention had already taken place (Table 2). The resulting parameter fits reflect this with a low worm burden that decreases year to year (0.084 and 0.002), with a highly aggregated distribution. The mf production and detection also decreases year to year (1.414 and 0.895); however, the CI for each is large, reflecting that the number of mf positive individuals in each year is extremely low, giving a greater degree of uncertainty in the parameter estimates.

**Table 2.** Estimated parameters (with 95% CI) for the 2 years surveys were conducted in Colombo and Gampaha, Sri Lanka

Year	mf detected in 100 μL blood per worm *α*	Mean worm burden *μ*	Worm aggregation *k_w_*
2004	1.414 (0.052–7.185)	0.084 (0.0427–1.410)	0.036 (0.008–0.226)
2005	0.895 (0.079–5.901)	0.002 (0.0–0.004)	0.081 (0.009–0.279)

mf: microfilariae.

The model was validated using both datasets by comparing the predicted probability of being both ICT positive and mf negative to the estimated values in the data. In all years there is close agreement between the data and the prediction (absolute errors for each year in Kenya are 0.2%, 0.4% and 0.2% and in Sri Lanka are 1.8% and 1.5%) (Table 3).

**Table 3.** Comparison of predicted probability of an individual receiving a positive immunochromatographic test (ICT) and negative microfilariae (mf) test with the estimates in the data for the two surveys

Location	Year	Predicted ICT positive, mf negative (%)	Data ICT positive, mf negative (%)
Malindi, Kenya	2002 (baseline)	16.5	16.7
	2003	16.1	16.5
	2004	13.2	13.0
Colombo and Gampaha, Sri Lanka	2004	2.1	3.9
	2005	1.7	0.2

**Figure 2.**
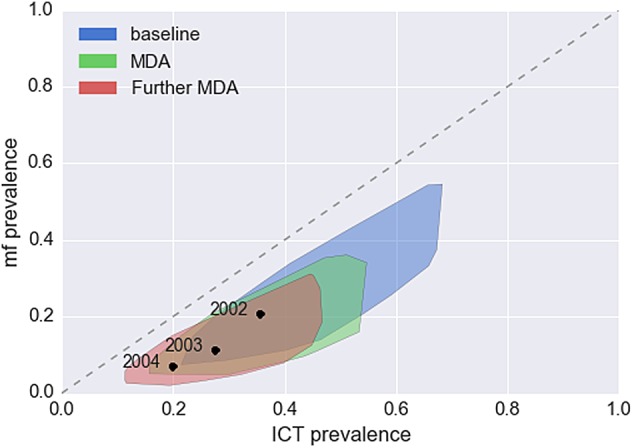
Estimated relationship between microfilariae (mf) prevalence and immunochromatographic test (ICT) prevalence under different mass drug administration (MDA) regimes, either at baseline; after one MDA treatment round and after two MDA treatment rounds. The estimated relationship was constructed by sampling from the posterior in each year from the Kenyan survey and using these parameters in the model to produce an mf–ICT relationship. The relationship found in the data is shown for 3 years as black dots.^15^

We subsequently used the model and these parameter estimates to investigate the predicted relationship between ICT and mf prevalence both pre-MDA and post-MDA for a range of scenarios. The baseline relationship across different settings shows high variance with the prevalence of mf below the prevalence of ICT.^10^ Post-MDA, the prevalence of mf is lowered while the prevalence of ICT remains high. After several rounds of MDA this leads to a flattened distribution, where the mf positives are fewer than the ICT positives. From the model, worm burden and aggregation can vary greatly between sites, leading to high variance in prevalence, and therefore ICT prevalence, whilst it is assumed that mf production is based upon the biology of the filarial worm and the presence of an MDA regime and hence will vary less across sites. By drawing worm burden, worm aggregation and mf production parameters from the estimated posterior in a pre and post-intervention setting, a distribution of mf-ICT prevalence values can be produced (Figure 2). This indicates that similar epidemiological parameters produce a wide variance relationship, where a wider range of antigenaemia prevalence can exist for narrower mf prevalence after subsequent MDA rounds.

**Figure 3.**
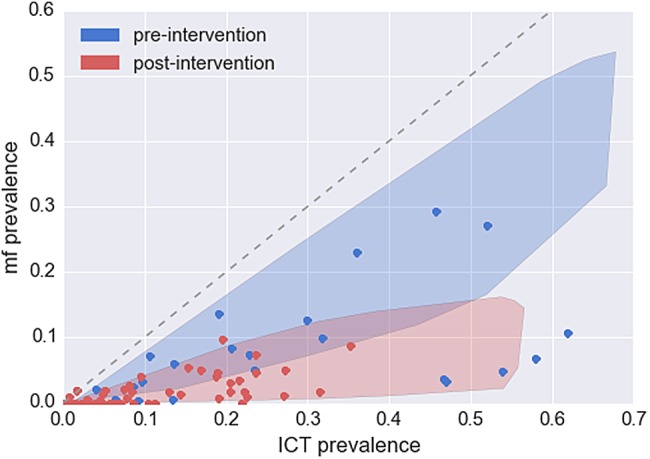
Comparison of microfilariae–immunochromatographic test (mf–ICT) prevalence relationship in a number of settings to the model predicted range with parameters estimated from the two fitted datasets. The Kenyan parameter range was used to produce the pre-intervention range, with a lower mean worm burden to account for a variety of endemic settings. A reduction in mean worm burden and mf output that corresponds with known drug regimen efficacies was applied to the range to produce the post-intervention area relationship. The relationship in pre and post-intervention setting were compared to a number of diverse studies where both antigenaemia and microfilaraemia were assessed.^21–24^ This illustrates how a combined reduction in mean worm burden combined with a higher reduction in mf output leads to the observed pattern in pre- and post-intervention settings.

With the model validated, the relationship derived from the fitted posteriors and known efficacies of MDA were compared to a number of datasets where the prevalence of ICT and mf were known. These data were stratified by whether diagnosis tests were taken before or after MDA^21–24^ (Figure 3). The model predictions match closely with the data, although there are notable outliers in the pre-intervention scenario.

## Discussion

In a recent article,^10^ the disparity between the pre- and post- intervention settings is highlighted. The authors found that a regression analysis was unable to provide a predictable relationship between the two measures of prevalence. Using minimal assumptions of population worm burden and mf distribution, we have constructed a statistical mechanistic model of the prevalence of antigenaemia (*p_ICT_*) and microfilaraemia (*p_mf_*) which explains these patterns. The model predicts a wide variance relationship between these prevalences in a site pre-intervention and a narrow relationship after several rounds of MDA, where antigenaemia is generally much higher than microfilaraemia in the population. Here, we first fitted and validated the model using a three-year sentinel site study in Malindi, Kenya before and during MDA. The fitted parameters show a drop in both the mf production and mean worm burden each year MDA occurred. This drop is consistent with other studies of MDA with diethylcarbamazine combined with albendazole.^25,26^ These new rates can then be used to estimate the relationship in subsequent years as MDA takes place and thus estimate the *p_mf_* from the *p_ICT_* alone. The updated parameters in subsequent years also give insight into the efficacy of MDA by providing an estimate of the reduction of mean worm burden and the reduction of mf production.

The early-intervention Kenyan dataset was contrasted with a late intervention dataset conducted in Western Sri Lanka. The fitted parameters also show a decline in mf production and mean worm burden after an MDA round, however the confidence interval on the mf production is large due to the fact that nearly all individuals surveyed were mf and ICT negative. This represents a difficulty in estimating the fecundity of individual worms when their numbers are extremely low and have a highly aggregated distribution.

A plausible relationship between the ICT and mf in pre- and post-intervention settings was also constructed by considering how the population worm distribution may vary geographically and between sites. Differences in immunity, history of infection, vector control, population density and lifestyle would all contribute to observed differences in mean worm burden, aggregation and mf production, which are parameter inputs for the model. The decline of mf production due to MDA reduces the overall mf production, whilst the mortality of adult worms is limited, thus leading to a high variance of *p_ICT_* with a low variance of *p_mf_*. The model, therefore, predicts the observed relationship in these two distinct settings and also provides a way of predicting the new prevalence relationship based upon the known mechanisms of filaricidal drugs used in MDA.

The validated model was compared to a number of datasets from a variety of settings. The broad pattern predicted by the model is reproduced by the data, with a greater reduction in mf prevalence than in the ICT prevalence. There are notable outliers in the pre-intervention setting. These regions have unusually low mf prevalence with high ICT prevalence. The model was fitted to an individual location, where it is assumed mf production varies with the application of MDA alone. Over more diverse regions other factors may need to be taken into account, such as history of infection, immune response of the population and time at which blood samples were taken.

### Limitations of the study

This model is based on a basic understanding of the distribution of adult worms and their detectability through antigenemia and mf output. The model does not currently include transmission and subsequently an understanding of how the parameters evolve in time is necessary to provide further predictions of the relationship going forward. The parameters are also likely to vary between settings as a number of factors change such as bite-risk heterogeneity, resulting in different distributions of adult worms. Using other forms of data would improve the estimate of these key parameters and provide further estimation of the relationship. Currently, both microfilaraemia and antigenaemia prevalence is required to calibrate the model and few studies consider both over subsequent years, before during and after an intervention campaign. The estimation also currently does not use the mf or antigen intensity. These data may help to further calibrate the model providing a more robust relationship between the prevalences. Importantly, our methodology does not allow a direct estimation of mf prevalence from ICT prevalence without some mf studies to inform the parameter estimation and it is unclear how transferable these studies are between different settings.

### Conclusions

We have shown using minimal assumptions of worm and mf distribution an explanation for the disparity between microfilaraemia and antigenaemia. The technique allows us to estimate key epidemiological parameters during a MDA programme as well as during post-MDA surveillance; these estimates can be compared to other diagnostic methods proposed for use in surveillance such as filarial antibodies.^27–29^ For example, ICT prevalence and mf prevalence data at baseline can be used to infer the mean worm burden and aggregation in a population in subsequent rounds, this combined with knowledge of drug efficacy in clearing mf can be used to calculate a credible interval for the prevalence of mf. This gives new insight in being able to determine the efficacy of an MDA programme based upon the reduction in mf production as opposed to the crude estimates of the prevalence alone. This insight would also be useful in preventing recrudescence by understanding how the mf prevalence may change in subsequent years if transmission has been broken or if worm burden is increasing in the population.
